# Fertilization and Colors of Plastic Mulch Affect Biomass and Essential Oil of Sweet-Scented Geranium

**DOI:** 10.1155/2014/828259

**Published:** 2014-03-16

**Authors:** Anderson de Carvalho Silva, Arie Fitzgerald Blank, Wallace Melo dos Santos, Paloma Santana Prata, Péricles Barreto Alves, Maria de Fátima Arrigoni-Blank

**Affiliations:** ^1^Department of Agronomic Engineering, Federal University of Sergipe, Avenida Marechal Rondon s/n, 49100-000 Sao Cristovao, SE, Brazil; ^2^Department of Chemistry, Federal University of Sergipe, Avenida Marechal Rondon s/n, 49100-000 Sao Cristovao, SE, Brazil

## Abstract

Sweet-scented geranium (*Pelargonium graveolens* L'Hér), a plant belonging to the Geraniaceae family, has medicinal and aromatic properties and is widely used in the cosmetic, soap, perfume, aromatherapy, and food industries. The aim of this study was to evaluate the influence of fertilization and the use of different colors of plastic mulch on sweet-scented geranium biomass and essential oil. Three colors of plastic mulch (black, white, and silver-colored) and a control without plastic mulch were assessed along with three fertilizers (20,000 L*·*ha^−1^ of cattle manure; 1,000 kg*·*ha^−1^ of NPK 3-12-6; and 20,000 L*·*ha^−1^ of cattle manure + 1,000 kg*·*ha^−1^ of NPK 3-12-6 fertilizer) and a control without fertilizer. The absence of a soil cover negatively influenced the agronomical variables, while coverage with plastic mulch was associated with increased biomass. The use of fertilizer had no effect on the evaluated agronomic variables. When cattle manure and NPK 3-12-6 were used together, combined with white or black plastic mulch, the highest yields of essential oil were obtained. For the silver-colored plastic mulch, higher amounts of essential oil (6,9-guaiadien) were obtained with mineral fertilizer.

## 1. Introduction

The* Pelargonium* (Geraniaceae) genus contains around 280 herbaceous, shrub, and subshrub species, including annuals and perennials that typically originated from southern Africa [1].* Pelargonium graveolens *L'Hér is one of the most important species in this genus and is an aromatic shrub species that can reach up to 1.3 m in height. This geranium is native to Cape Province, South Africa, and is grown in many regions worldwide, including the Reunion Islands, Algeria, southern France, Spain, Morocco, Madagascar, Congo, and Russia, for the production of its essential oil [2–6]. The geranium essential oil is an important material for the cosmetic, soap, perfume, aromatherapy, and food industries [2, 7–10]. It is also used in popular medicines against dysentery, diarrhea, colds, and lung infections [3, 11].

The production of essential oils in plants may be altered by environmental or ontogenic factors, such as the physical-chemical characteristics of the soil, moisture, temperature, developmental stage, and phenology [12]. These factors have therefore been evaluated to determine how they influence the production of volatile compounds in medicinal and aromatic species [8, 13–15].

Soil composition is one of the determining factors for crop establishment and may be altered to meet the nutritional needs of plants. Agricultural inputs, namely, chemical or organic fertilizers, can be used for this purpose. Organic fertilizers naturally reset nutritional characteristics and rely on the production of organic matter by decomposition, which is an important source of nutrients for plants, microflora, and terrestrial fauna. In the soil, organic matter provides nutrients and has an important role as a conditioner of the physical, chemical, and biological properties of the soil [16]. The traditional cultivation of geranium combines chemical fertilizer and organic matter inputs. With respect to essential oil production, the cultivation of* P. graveolens *is more profitable when the crop is grown in soils rich in organic matter [17].

For the use of proper growth conditions and increase of the production of active constituents it is necessary to develop agricultural technologies for a correct cultivation of aromatic plants [16, 18]. Mulching is a common technology used in the cultivation of medicinal and aromatic species to increase the production of active ingredients [19]. Among other benefits, the use of mulch regulates surface radiation, promotes vegetative growth and productivity, increases insect repellence, controls weeds, decreases water loss by evaporation, and facilitates harvesting [20].

The aim of this study was to evaluate the effect of organic-mineral fertilizers and soil coverage with plastic mulch on sweet-scented geranium (*P. graveolens*) biomass and essential oil.

## 2. Materials and Methods 

### 2.1. Plant Materials

Plants of sweet-scented geranium (*Pelargonium graveolens* L'Hér) genotype UFS-PEL001 were propagated by cuttings. The plants were maintained in 1500 cm^3^ plastic bags containing a 1 : 1 mixture of soil and sand as substrate for 30 days and then transplanted to the field. Genotype exsiccates were deposited in the ASE Herbarium of the Federal University of Sergipe (UFS), under the record number ASE-14844.

### 2.2. Experimental Procedure

The assay was conducted at the “Campus Rural da UFS” Research Farm, located in São Cristóvão, Sergipe State, Brazil, from 2008 to 2009. The climate of the region is tropical semiarid, and the soil is classified as Red-Yellow Argisol with low fertility. The chemical characteristics of the soil are as follows: pH = 5.4 in water; *P* = 2.3 mg·dm^−3^; K (Mehlich 1) = 0.09 cmolc·dm^−3^; Ca + Mg [21] = 2.70 cmolc·dm^−3^; and organic matter = 21.1 g·dm^−3^.

A randomized block design was used for the experiment, with three replicates in a split-plot design. In the plots, three different colored plastic mulches (black, white, and silver-colored) were tested in addition to the control with no plastic mulch. In the subplots, three fertilizer treatments (20,000 L·ha^−1^ of cattle manure; 1,000 kg·ha^−1^ of NPK 3-12-6; and 20,000 L·ha^−1^ of cattle manure + 1,000 kg·ha^−1^ of NPK 3-12-6) were tested in addition to the control without fertilizer. Fertilizer was applied 15 days before transplantation of the plants to the field.

The spacing between rows and between plants was 50 cm. The beds were 90 cm wide, and each plot contained four plants cultivated in two rows. A drip irrigation system was used. Planting in the field was performed on May 28, and the first harvest was realized on August 26. Subsequent harvests were realized 90 days apart.

At each harvest, the height and canopy diameter of each plant was measured before cutting the plants and after cutting the fresh weight was measured. To determine the moisture content, three 100 g fresh leaf samples were dried in an oven dryer at 105°C until constant weight.

### 2.3. Essential Oils

The essential oils of the fresh leaves were obtained by steam distillation using a Clevenger apparatus [22] for 2 h and 40 min [23]. The content percentage was estimated using the dry matter weight (v/m) of the three 100 g leaf samples.

### 2.4. Analysis of Essential Oils

The analysis of the essential oil chemical composition was performed in a gas chromatograph coupled to a mass spectrometer (GC-MS) (Shimadzu, model QP 5050A) equipped with an AOC-20i autoinjector (Shimadzu) and a fused-silica capillary column (5%-phenyl-95%-dimethylpolysiloxane, 30 m × 0.25 mm id., 0.25 *μ*m film, J&W Scientific). Helium was used as the carrier gas at a flow rate of 1.2 mL/min. The temperature program was as follows: 50°C for 1.5 min, temperature increase at 4°C/min until reaching 200°C, temperature increase at 15°C/min until reaching 250°C, and 250°C for 5 min. The injector temperature was 250°C, and the detector (or interface) temperature was 280°C. The injection volume of ethyl acetate was 0.5 *μ*L, the partition rate of the injected volume was 1 : 87, and the column pressure was 64.20 kPa. The mass spectrometer conditions were as follows: ionic capture detector impact energy of 70 eV and scanning speed 0.85 scan/s from 40 to 550 Da.

Quantitative analysis of the chemical constituents was performed by flame ionization gas chromatography (FID), using a Shimadzu GC-17A (Shimadzu Corporation, Kyoto, Japan) instrument, under the following operational conditions: capillary ZB-5MS column (5% phenyl-arylene-95%-dimethylpolysiloxane) and fused-silica capillary column (30 m × 0.25 mm i.d. × 0.25 *μ*m film thickness) from Phenomenex (Torrance, CA, USA), under same conditions as reported for the GC-MS. Quantification of each constituent was estimated by area normalization (%). Compound concentrations were calculated from the GC peak areas and they were arranged in order of GC elution.

The essential oil components were identified by comparing their mass spectra with the available spectra in the equipment database (NIST05 and WILEY8). Additionally, the measured retention indices were compared with those in the literature [24]. The relative retention indices (RRI) were determined using the Vandendool and Kratz [25] equation and a homologous series of *n*-alkanes (C_8_-C_18_) injected under the chromatography conditions described above.

### 2.5. Statistical Analysis

We analyzed the following variables during the 12-month period: survival (%), plant height (cm), canopy diameter (cm), leaf fresh weight (g·plant^−1^), leaf moisture (%), essential oil content (%), and yield (mL·plant^−1^). The content (%) of the following chemical constituents was determined: citronellol, geraniol, citronellyl formate, linalool, 6,9-guaiadien, geranyl formate, geranial, and isomenthone.

The means of the variables were subjected to the analysis of variance *F* test and were compared using the Scott-Knott test at 5% probability.

## 3. Results

### 3.1. Agronomical Variables

The use of plastic mulch is common in vegetable production, but the effect of this mulching approach has not been widely studied in medicinal and aromatic plants.

The results from the present study indicate that the interaction between the type of plastic mulch and the type of fertilizer was significant only for the yield of essential oil. The use of cattle manure alone or cattle manure + NPK 3-12-6, combined with white plastic mulch, produced a higher essential oil yield than did the same treatment with black or silver-colored plastic mulch (Table 1). As for the other variables, the lack of plastic mulch significantly reduced survival and leaf fresh weight and the essential oil yield of the plants from the first two harvests. For the third and fourth harvests, reductions were observed for all the variables (Table 2).

For the different fertilization treatments, there were no significant differences observed for the assessed variables (Table 1).

### 3.2. Essential Oils

The contents of several of the analyzed compounds varied significantly when different types of fertilizers were used. In addition, there were variations in compound content when the plants were grown under different colored plastic mulches (Table 3).

There was an interaction between the various factors of the contents of citronellol, geraniol, 6,9-guaiadien, geranial, and isomenthone. The citronellyl formate content was only affected by colors of plastic mulch. The linalool and geranyl formate contents were not affected by types of fertilizers and colors of plastic mulches (Table 3). Significant differences in citronellol content were observed for the different fertilizer treatments when no plastic mulch was used. In this case, the use of cattle manure as fertilizer led to an increased content of citronellol. There were significant differences in geraniol content for the different colors of plastic mulch with the NPK (3-12-6) and cattle manure + NPK (3-12-6) fertilizer treatments; greater geraniol content was observed when no plastic mulch was used compared to the other plastic mulch treatments. The citronellol content was greater when cattle manure was used as fertilizer, while geraniol production increased when NPK (3-12-6) was used.

The lowest contents of 6,9-guaiadien were observed with the silver-colored plastic mulch using no fertilizer or cattle manure for fertilizing (Table 3). The production of geranial was strongly reduced by two particular combinations: the use of a black plastic mulch with cattle manure and the use of a silver-colored plastic mulch with cattle manure + NPK 3-12-6 (Table 3). These observations highlight the negative influence of cattle manure combined with the plastic mulches on the geranial compound.

Significant differences were also observed for citronellyl formate content, which was reduced when the silver-colored plastic mulch was combined with cattle manure or without fertilizer. The isomenthone content was lowest when NPK 3-12-6 was combined with no plastic mulch and when cattle manure was combined with the black plastic mulch.

## 4. Discussion

The present evaluation of different colored plastic mulches demonstrates the effectiveness of mulching for geranium production in light of the fact that the control treatment (without mulch) resulted in lower values for almost all of the agronomic variables assessed. Plant development is certainly influenced by factors affected by the use of plastic mulch, such as moisture retention, reduced competition for light and nutrients due to the absence of weeds, a reduction of pests, and an increase in the surface temperature of the soil [26].

The findings of similar studies confirm these benefits, including the study of [27], which demonstrated a reduction in the productivity of cantaloupe when no plastic mulch was used. In experiments investigating the use of plastic mulch on potato crops, plants were found to emerge earlier, achieving greater height and tuber volume, when a plastic mulch was used on the soil [28, 29]. In a study assessing five different plastic mulch colors (black, white, red, silver, and blue) on an okra crop, the greatest yield was obtained with the silver-colored plastic mulch, followed by the blue plastic mulch, while the control treatment with no mulch produced the lowest yield [30].

Moreover, studies evaluating the effects of other factors associated with soil coverage, such as changes in the soil microclimate and the incidence of agricultural pests, demonstrate how the use of plastic mulch can be advantageous and economical for producers [31–33].

In* Melissa officinalis* L. soil coverage, with either pine needles or black plastic mulch, improved the biomass and essential oil yields [34]. In a related study in patchouli (*Pogostemon cablin *(Blanco) Benth.), the use of organic mulch for soil coverage improved the yield, amount, and quality of the patchouli essential oil [35]. In* P. graveolens*, an absence of soil coverage was associated with reduced yield and essential oil content along with changes in the contents of the major components of the essential oil [18]. In the present study, different fertilizer treatments did not influence the cultivation of geranium; this finding is probably due to the low nutritional requirements of the crop and its tolerance of acidic soils. It is likely that the existing nutrients in the soil sufficiently supported the crop during the evaluation period. On the other hand, when different levels of nitrogen fertilizer were tested in* P. graveolens*, significant differences were observed for the biomass and essential oil yield and content [18]. In a related study in* Ocimum basilicum* L. the use of organic-mineral fertilizer did not significantly influence the development of the plants or the production of essential oil [36]. Gopichand et al. [37] similarly observed that different levels of organic fertilizer did not significantly affect the development, yield, and essential oil content of* Curcuma aromatica*.

The results of the present study suggest that the interaction observed among the various factors and the changes in the contents of the major components of the geranium essential oil were due to the benefits provided by the plastic mulch: the temperature and humidity of the soil were maintained, and the resources necessary for the biosynthesis of secondary metabolites were available to the plants. The high contents of the essential oil and its major components in treatments where a plastic cover was used are in accordance with the data found in the literature.

## 5. Conclusions

The absence of a soil cover negatively influenced the agronomical variables, while coverage with plastic mulch was associated with increased biomass. When cattle manure and NPK 3-12-6 were used together, combined with white or black plastic mulch, the highest yields of essential oil were obtained. For the silver-colored plastic mulch, higher amounts of essential oil (6,9-guaiadien) were obtained with mineral fertilizer.

## Figures and Tables

**Table 1:** The effect of different colors of plastic mulch and fertilizers on content and yield of sweet-scented geranium essential oil.

Fertilizer	Colors of plastic mulch			
	Control	White	Black	Silver-colored
	Essential oil content (%)			
Control	1.30^aA^	1.42^aA^	0.87^aA^	1.16^aA^
Cattle manure	1.32^aA^	1.51^aA^	1.15^aA^	0.96^aA^
NPK 3-12-6	1.23^aA^	1.14^aA^	1.16^aA^	1.26^aA^
Cattle manure + NPK 3-12-6	1.30^aA^	1.25^aA^	1.08^aA^	1.16^aA^

	Essential oil yield (mL·plant^−1^)			
Control	0.32^aA^	0.49^aA^	0.26^aA^	0.52^aA^
Cattle manure	0.21^aB^	1.04^aA^	0.42^aB^	0.59^aB^
NPK 3-12-6	0.21^aA^	0.71^aA^	0.45^aA^	0.62^aA^
Cattle manure + NPK 3-12-6	0.18^aB^	1.02^aA^	0.64^aB^	0.97^aA^

Lowercase letters indicate differences within columns, and uppercase letters indicate differences within rows. Values followed by the same letter are not statistically different based on the Scott-Knott test (*P* ≤ 0.05).

**Table 2:** The effect of different colors of plastic mulch and fertilizers on the survival and leaf fresh weight of sweet-scented geranium.

Survival (%)				
Fertilizer	Colors of plastic mulch			
	Control	White	Black	Silver colored
	First harvest			
Control	58.33^Aa^	100.00^Aa^	100.00^Aa^	100.00^Aa^
Cattle manure	58.33^Aa^	91,66^Aa^	100.00^Aa^	100.00^Aa^
NPK 3-12-6	58.33^Aa^	100.00^Aa^	100.00^Aa^	100.00^Aa^
Cattle manure + NPK 3-12-6	25.00^Bb^	100.00^Aa^	100.00^Aa^	100.00^Aa^

	Second harvest			
Control	58.33^Aa^	91.66^Aa^	100.00^Aa^	100.00^Aa^
Cattle manure	58.33^Aa^	100.00^Aa^	100.00^Aa^	100.00^Aa^
NPK 3-12-6	58.33^Aa^	100.00^Aa^	100.00^Aa^	100.00^Aa^
Cattle manure + NPK 3-12-6	16.66^Ba^	100.00^Aa^	100.00^Aa^	100.00^Aa^

	Third harvest			
Control	58.33^Aa^	100.00^Aa^	100.00^Aa^	100.00^Aa^
Cattle manure	58.33^Aa^	91.66^Aa^	100.00^Aa^	100.00^Aa^
NPK 3-12-6	41.66^Ba^	100.00^Aa^	100.00^Aa^	100.00^Aa^
Cattle manure + NPK 3-12-6	16.66^Bb^	91.66^Aa^	100.00^Aa^	100.00^Aa^

	Fourth harvest			
Control	25.00^Bb^	91.66^Aa^	100.00^Aa^	100.00^Aa^
Cattle manure	33.33^Ba^	83.33^Aa^	100.00^Aa^	91.66^Aa^
NPK 3-12-6	33.33^Ba^	83.33^Aa^	100.00^Aa^	100.00^Aa^
Cattle manure + NPK 3-12-6	25.00^Bb^	91.66^Aa^	50.00^Bb^	100.00^Aa^

Leaf fresh weight (g·plant^−1^)				
Fertilizer	Colors of plastic mulch			
	Control	White	Black	Silver colored

	First harvest			
Control	107.65^aA^	164.33^bA^	150.07^aA^	303.09^aA^
Cattle manure	70.83^Ba^	346.65^Aa^	179.76^Ba^	319.15^Aa^
NPK 3-12-6	78.51^Ba^	303.38^Aa^	148.52^Ba^	248.25^Aa^
Cattle manure + NPK 3-12-6	62.53^Ba^	374.98^Aa^	261.36^Aa^	363.86^Aa^

	Second harvest			
Control	106.65^Aa^	311.32^Aa^	196.28^Aa^	372.15^Aa^
Cattle manure	82.21^Aa^	332.41^Aa^	232.34^Aa^	404.18^Aa^
NPK 3-12-6	65.17^Ba^	368.68^Aa^	215.04^Ba^	359.23^Aa^
Cattle manure + NPK 3-12-6	23.28^Ba^	299.97^Aa^	272.50^Aa^	305.56^Aa^

	Third harvest			
Control	141.66^Ba^	425.00^Aa^	187.50^Ba^	389.83^Aa^
Cattle manure	137.50^Ba^	555.00^Aa^	316.66^Ba^	482.44^Aa^
NPK 3-12-6	136.11^Aa^	434.16^Aa^	326.66^Aa^	334.71^Aa^
Cattle manure + NPK 3-12-6	21.66^Ba^	456.39^Aa^	324.16^Aa^	451.29^Aa^

	Fourth harvest			
Control	23.33^Aa^	23.33^Aa^	141.66^Aa^	269.16^Aa^
Cattle manure	55.00^Aa^	55.00^Aa^	183.99^Aa^	207.50^Aa^
NPK 3-12-6	63.33^Aa^	63.33^Aa^	203.33^Aa^	138.33^Aa^
Cattle manure + NPK 3-12-6	150.00^Aa^	150.00^Aa^	216.66^Aa^	289.16^Aa^

Lowercase letters indicate differences within columns, and uppercase letters indicate differences within rows. Values followed by the same letter are not statistically different based on the Scott-Knott test (*P* ≤ 0.05).

**Table 3:** The effects of different colors of plastic mulch and fertilizers on contents of citronellol, geraniol, citronellyl formate, linalool, 6,9-guaiadien, geranyl formate, geranial, and isomenthone in sweet-scented geranium essential oil.

Fertilizer	Colors of plastic mulch			
	Control	White	Black	Silver-colored
	Citronellol (%) (RRI = 1223)			
Control	24.68^bB^	28.18^aA^	26.95^aB^	30.42^aA^
Cattle manure	29.44^aA^	29.46^aA^	31.22^aA^	28.72^aA^
NPK 3-12-6	25.14^bB^	30.56^aA^	28.73^aA^	29.00^aA^
Cattle manure + NPK 3-12-6	23.68^bB^	29.44^aA^	29.61^aA^	30.64^aA^

	Geraniol (%) (RRI = 1249)			
Control	20.85^aA^	17.99^aA^	19.88^aA^	19.04^aA^
Cattle manure	18.99^aA^	19.39^aA^	20.63^aA^	20.28^aA^
NPK 3-12-6	21.86^aA^	17.80^aB^	18.21^aB^	16.66^aB^
Cattle manure + NPK 3-12-6	24.72^aA^	18.99^aB^	18.99^aB^	18.49^aB^

	Citronellyl formate (%) (RRI = 1271)			
Control	11.16^aA^	10.97^aA^	11.40^aA^	12.00^bA^
Cattle manure	11.69^aA^	11.67^aA^	12.15^aA^	10.69^bA^
NPK 3-12-6	11.11^aA^	11.87^aA^	12.16^aA^	14.28^aA^
Cattle manure + NPK 3-12-6	9.54^aA^	11.69^aA^	12.52^aA^	14.61^aA^

	Linalool (%) (RRI = 1095)			
Control	12.36^aA^	11.80^aA^	10.00^aA^	10.89^aA^
Cattle manure	10.31^aA^	11.42^aA^	10.06^aA^	8.99^aA^
NPK 3-12-6	9.95^aA^	13.08^aA^	13.55^aA^	12.82^aA^
Cattle manure + NPK 3-12-6	11.63^aA^	10.31^aA^	12.66^aA^	9.90^aA^

	6,9-guaiadien (%) (RRI = 1442)			
Control	6.19^aA^	5.95^aA^	5.74^aA^	4.39^bB^
Cattle manure	6.02^aA^	4.93^aA^	4.84^aA^	4.56^bA^
NPK 3-12-6	5.93^aA^	5.46^aA^	5.68^aA^	6.10^aA^
Cattle manure + NPK 3-12-6	5.07^aA^	6.02^aA^	5.04^aA^	5.87^aA^

	Geranyl formate (%) (RRI = 1298)			
Control	3.50^aA^	3.48^aA^	4.10^aA^	4.12^aA^
Cattle manure	3.63^aA^	3.73^aA^	3.55^aA^	3.79^aA^
NPK 3-12-6	3.25^aA^	3.12^aA^	3.91^aA^	4.62^aA^
Cattle manure + NPK 3-12-6	3.39^aA^	3.63^aA^	3.29^aA^	5.64^aA^

	Geranial (%) (RRI = 1264)			
Control	1.12^aA^	1.29^aA^	1.33^aA^	1.12^aA^
Cattle manure	1.18^aA^	0.79^aA^	0.00^bB^	1.26^aA^
NPK 3-12-6	1.18^aA^	1.13^aA^	1.20^aA^	1.08^aA^
Cattle manure + NPK 3-12-6	1.12^aA^	1.19^aA^	1.16^aA^	0.30^bB^

	Isomenthone (%) (RRI = 1158)			
Control	1.23^aA^	1.40^aA^	1.24^aA^	1.37^aA^
Cattle manure	1.18^aA^	1.18^aA^	0.93^bA^	1.24^aA^
NPK 3-12-6	0.99^aB^	1.40^aA^	1.31^aA^	1.31^aA^
Cattle manure + NPK 3-12-6	1.18^aA^	1.18^aA^	1.25^aA^	1.32^aA^

RRI: relative retention index.

Lowercase letters indicate differences within columns, and uppercase letters indicate differences within rows. Values followed by the same letter are not statistically different based on the Scott-Knott test (*P* ≤ 0.05).
